# Balloon-Assisted Colonoscopy after Incomplete Conventional Colonoscopy—Experience from Two European Centres with A Comprehensive Review of the Literature

**DOI:** 10.3390/jcm9092981

**Published:** 2020-09-15

**Authors:** Robertson Alexander R, Koulaouzidis Anastasios, Yung Diana E, Fraser Christopher, Nemeth Artur, Trimble Kenneth, Toth Ervin, Plevris John N, Wurm Johansson Gabriele

**Affiliations:** 1 Department of Gastroenterology, Western General Hospital, Edinburgh EH4 2XU, UK; Alexander.Robertson@nhslothian.scot.nhs.uk; 2 Endoscopy Unit, The Royal Infirmary of Edinburgh, Edinburgh EH16 4SA, UK; chris.fraser@nhs.net; 3 Centre for Liver & Digestive Disorders, The Royal Infirmary of Edinburgh, Edinburgh EH16 4SA, UK; diana.e.yung@gmail.com (Y.D.E.); ken.trimble@nhslothian.scot.nhs.uk (T.K.); J.Plevris@ed.ac.uk (P.J.N.); 4 Department of Gastroenterology, Skane University Hospital, Lund University, 205 02 Malmö, Sweden; artur.nemeth@med.lu.se (N.A.); ervin.toth@med.lu.se (T.E.); gabriele.WurmJohansson@skane.se (W.J.G.)

**Keywords:** balloon-assisted endoscopy, colonoscopy, caecal intubation, intervention

## Abstract

Background: Conventional colonoscopy (CC) allows access for colonic investigation and intervention; in the small group in whom CC is unsuccessful alternative imaging is often sufficient. There remains a subset, however, requiring full colonic visualisation or intervention. Balloon-assisted colonoscopy (BAC) gives a further option when access is difficult. *Aims:* This study aims to present the experience with BAC of two European tertiary referral centres. *Methods:* Procedures were carried out under local protocol over 15-years (2006–2020). Markers of procedural quality such as caecal intubation, complications and comfort were retrospectively compiled and analysed. Published evidence was summarised for comparison. *Results:* 122 procedures were undertaken, with polyps the most frequent indication and 90.2% having at least one previously incomplete CC. Features associated with difficult colonoscopy were common, including intraabdominal surgery (32.0%). 92.6% reached the caecum; completion was higher (96.3%) in those failing CC due to discomfort and lower in those failing due to anatomical difficulties (90.7%) or previous surgery (84.6%). Mean time to the caecum was 20.9 minutes and mean midazolam and fentanyl doses were 2.6 mg and 49.9 µg with low discomfort scores. *Conclusion(s):* Balloon-assisted colonoscopy is successful in >90% of patients, is well-tolerated, and is safe.

## 1. Introduction

The flexible endoscope has been in evolution since the 1950s and today is established as the principal mode for investigation and intervention of the colonic and terminal ileal mucosa [1,2]. Despite ongoing development in colonoscope design and improvements in endoscopic technique(s), there remains a subset of patients in whom complete colonoscopy proves challenging. A prerequisite for independent colonoscopy practice across the globe is that an endoscopist’s caecal intubation rate should be at least 90% [3,4]. In expert hands, the expectation would be that the number of incomplete procedures is reduced to a very small subset of difficult cases [5]. To complete the examination of the ileocolonic area, when therapy is not required, either computed tomography colonoscopy (CTC) or colon capsule endoscopy (CCE) is often adequate [6,7].

**Figure 1:**
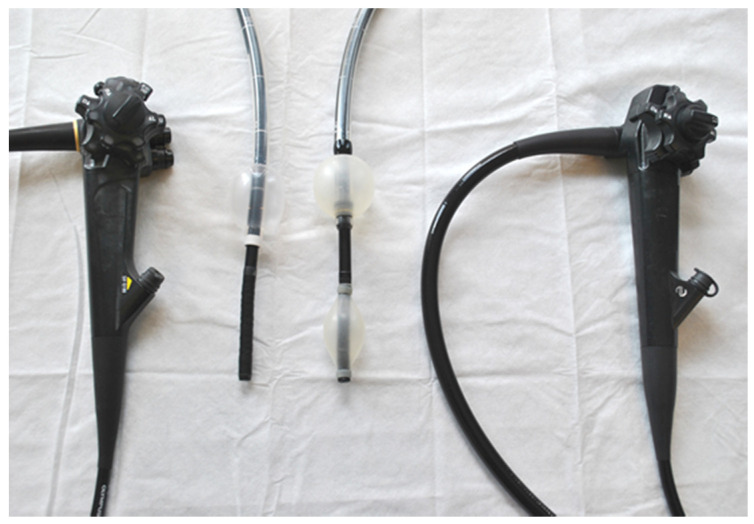
A single balloon endoscope (left) and double balloon endoscope (right).

Nevertheless, there will always be a small group of patients with challenging colons in whom complete colonoscopy is necessary for visualisation or therapy. In this subgroup, balloon-assisted colonoscopy (BAC) is an option. This technique allows the colon to be gripped by inflatable balloons and the colon shortened for ease of navigation and tip control stability, Figure 1. The technique follows the principles of double-balloon enteroscopy (DBE), which was developed to allow deep exploration of the small bowel by Yamamoto et al. [8], with simplification to a single-balloon (SBE) system following [9]. The utility of these systems for completion of long and difficult colonoscopies was quickly realised [10] with a couple of studies [11,12] also presenting a comprehensive review of the literature, which shows BAC to be safe and effective. In these centres, the first line of investigation following failed colonoscopy is CT colonography (CTC) and CCE. When it is felt likely that therapy is likely to be required however BAC becomes the preferred option.

This manuscript aims to present the practice of using BAC in two European tertiary referral centres when conventional colonoscopy has either been incomplete, or unable to provide a stable platform for therapy, and present a summary of the evidence available internationally to support our findings.

## 2. Materials and Methods

Balloon-assisted colonoscopies performed in Edinburgh (Scotland) and Malmö (Sweden) between March 2006 and March 2020 were retrospectively reviewed. Procedures during this time were carried out as per departmental protocol(s). Double-balloon procedures were performed with Fujinon (Tokyo, Japan) endoscopes EC450-B15, EC450-LP5, EN450-T5, EC530-A131, EN580-T, EC600WL and single-balloon procedures with Olympus Optical Co. (Tokyo, Japan) endoscope SIF-Q180. Data regarding procedure details and outcomes were retrieved from endoscopy reporting systems on patients undergoing BAC. Patient records were retrospectively reviewed for demographics, medical history, indication(s) for colonoscopy, reason for failed conventional colonoscopy, indication for BAC, the type of endoscope, caecal intubation, findings, intervention(s) performed, sedation used, complications, and finally patient comfort during the procedure. These data were anonymised and securely stored for analysis.

### 2.1. Statistics

Data were collated, analysed and graphed through Microsoft Excel (Microsoft Corporation, WA, USA). Results are descriptive with values expressed as mean (±SD) or median (range). Student’s t-test was used to compare means with the level of statistical significance *p* < 0.05.

### 2.2. Ethics 

This study used routinely collated data and conducted as a service evaluation. Anonymised data were stored securely, with methodology in compliance with the Declaration of Helsinki.

## 3. Results

### 3.1. Cohort Demographics

A total of 122 procedures (66 in female patients) between March 2006 and March 2020 were included and reviewed. Of those, 110 used a double-balloon endoscope (DBC), with 12 using single-balloon (SBC). All SBCs were performed at Skane University Hospital in Malmö. All procedures were performed by senior endoscopists, this included five individuals in Malmö and three in Edinburgh. The endoscopists were all certified as independent in colonoscopy with life experience of >1000 procedures. The median age of patients at the time of procedure was 66.5 (20–89) years. Indications for the procedures are listed in Table 1.

### 3.2. Previous Abdominal Surgery and Other Comorbidities

Of the patients, 32.0% (39/122) had had previous abdominal surgery; these are listed in Table 2. Furthermore, the presence of significant medical comorbidity was common, Table 2.

### 3.3. Reason for Failure of Conventional Colonoscopy

Incomplete conventional colonoscopy prior to BAC was recorded in 90.2% (110/122). In 12 (9.8%) cases, although the caecum was reached, polypectomy was not possible due to unstable position, thus requiring the procedure to be repeated. Overall, 63.1% (77/122) had only one previous incomplete procedure, 15.6% (19/122) had two, and 11.5% (14/122) had more than two incomplete procedures prior to BAC. In individuals with previous failed procedures (*n* = 110) the reasons included anatomical (long/looping or redundant/capacious colon *n* = 63, diverticula *n* = 2, fixed/angulated area *n* = 10), patient discomfort (*n* = 27), unclear (*n* = 6) or small-bowel pathology (*n* = 2).

### 3.4. Success Rate of BAC and Reasons for BAC Failure

BAC had a caecal intubation rate of 92.6%. Nine BACs were incomplete due to poor bowel preparation, significant pathology encountered or anatomy. In total, 8.3% (1/12) of the SBC and 7.3% (8/110) of the DBC procedures were failed. None were due to complications or severe discomfort to the patient. Six of the failed BACs were in patients with previous abdominal surgery. As such, the caecal intubation rate of BAC in those with previous abdominal surgery was lower at 84.6% (33/39). The operations and comorbidities of those having failed BAC were varied. For patients who had an incomplete initial colonoscopy due to discomfort caecal intubation with BAC was achieved in 96.3% (26/27). Caecal intubation was 90.7% (68/75) in those failing initial colonoscopy because of angulation, diverticula or long colon (six BAC failures had been in those labelled initially as long capacious colon and one angulation). Characteristics of those with incomplete BAC (*n* = 9) are shown in Table 3.

### 3.5. Findings and Therapies Given

The largest group requiring BAC were for investigation and treatment of polyps or to improve stability to safely enable polypectomy and this was the most common finding in this cohort (*n* = 67). In this group, 89.6% (60/67) had polypectomy ± argon plasma coagulation (APC), tattoo or clips during BAC procedure. Other primary findings are shown in Table 4.

### 3.6. Time to Caecum, Comfort and Complications

The time taken to reach the caecum was recorded in 31 procedures with a mean of 20.9 (±13.5) and median of 19 (4–60) minutes. Five individuals required propofol or general anaesthetic (GA) for their BAC procedure (117 had conscious sedation ± analgesia or neither). Those having conscious sedation with midazolam (*n* = 96) had a mean dose of 2.6 mg (±1.9 mg). A total of 77 patients had received fentanyl for their procedure at mean dose of 49.9 µg (±45.0 µg). There was no significant difference between the dose of midazolam given between those having SBC or DBC (*p* = 0.34), although none of the SBC patients had fentanyl.

Discomfort scores were subjectively reported by the endoscopist and recorded for 82 procedures. No discomfort reported in 36.6% (30/82), mild in 47.6% (39/82), moderate in 14.6% (12/82), significant in 1.2% (1/82) and none for severe. The only complication reported was a vasovagal attack during polypectomy.

## 4. Discussion

Even the best endoscopists will inevitably encounter a colon which they cannot fully traverse with conventional colonoscopy, irrespective of the tips and tricks of the trade [5]. In such cases, there are several options available, and the decision should be tailored to the individual patient. In procedures with diagnostic intention less invasive complimentary imaging techniques such as CTC and/or CCE can be considered [6]. For those who require intervention, however, an invasive procedure is still required.

We present herein the experience of using BAC, in two European tertiary referral centres, to address this issue, which represents the largest single study published so far. The most frequent indication (43%) in this cohort was providing access and stable instrument tip position for accurate polyp assessment and safe polypectomy in difficult colons. Previous studies have frequently sited polypectomy as the most common indication for DBC, see Table 5. In this study, most patients successfully underwent polypectomy of often large or difficult lesions. The reasons for no polypectomy taking place included the polyp being isolated and unresectable or post inflammatory. Others had colitis or incomplete procedure (due to obstructing pathology, poor prep) or no polyp being found. In this cohort several patients had caecal intubation on initial colonoscopy. Although the caecum had been reached, these procedures were still not adequate to provide the required intervention and so repeat with BAC allowed a safer, more stable position from which polypectomy could be achieved. This in our opinion is a strength and strong indication for BAC. An informative tabulated summary of published evidence on the use BAC is also presented, Table 5.

The relatively low number of procedures (122 procedures between 2006 and 2020) indicates that BAC is reserved for a small percentage of patients. In our centres, BAC is primarily reserved for those who are likely to require therapy, as CT colonography and CCE provide adequate diagnostic information in most. Despite this, there were 29 patients who did not have pathology seen on their BAC. When an invasive procedure is required, other options such as axis-shortening techniques including underwater/water immersion colonoscopy for longer, looping colons, or deep sedation (propofol)/general anaesthesia for anxiety or tenderness are more frequently employed. Over the course of a long study period, there were relatively few patients requiring BAC, but this included assessment of IBD or abnormal imaging, who were deemed likely to require biopsies or requiring haemostatic treatment, which was most frequently APC. In our study, there was no difference in the completion rate of DBC and SBC, which is in accordance to the previous publications, showing that the outcomes are similar using double and single-balloon colonoscopy in patients with previously failed or difficult colonoscopy. [10,11]. This suggesting that the choice of instrument would depend on local availability or expertise.

The patients seen were elderly and frequently female, with a high incidence of cardiorespiratory comorbidity. They were also frequently observed to have had previous abdominal surgery (in roughly a third). These being features associated with a more difficult colonoscopy and initial procedures were failed in most due to either long capacious colons or angulation. BAC in these patients had a completion rate of 90.7%, with failure of BAC most often associated with previous abdominal surgery. Tolerance was good following previous experience of discomfort during initial colonoscopy, with 70.6% (12/17 of those with recorded discomfort scores) undergoing conscious sedation with discomfort score of ‘mild’ or ‘no discomfort’. The caecal intubation rate was high, at 96.3% in those previously failing due to discomfort. Sedation practice during this time has also changed. Although none of the cases in Edinburgh used propofol or GA, it was introduced in Malmö in 2012. Five patients required propofol or GA for their procedure and these patients were spread over the course of the study period, both pre and post 2012. Many patients managed BAC without any analgesia or sedation and those given IV medication tolerated the procedure with a mean dose of midazolam of 2.6 mg and fentanyl of 49.9 mg.

There are several limitations of this study. It is retrospective in design and without a comparison group, making the significance of the percentages difficult to extrapolate. There is also a prolonged study period across two northern European centres, during which time, equipment and training have improved. Although the addition of propofol as an option in Malmö may have also reduced the failure of conventional colonoscopy, half of those requiring BAC following procedures failed due to discomfort were after 2012. The patients also varied, but as older, comorbid patients they are usually excluded from trials. As such, this study, which includes these complicated patients with failed and difficult colonoscopies who require intervention, has strengths. It represents a difficult real-world situation which is frequently encountered in clinical practice and shows that in the vast majority BAC provides a solution. A recent meta-analysis of balloon-assisted colonoscopy in patients with difficult or incomplete procedures included 667 patients across 18 studies [12]. This paper represents the largest study of which we are aware at the time of writing and we would agree with the meta-analysis’ conclusion that major centres should have balloon assisted colonoscopy available as a rescue technique.

In patients requiring a complete diagnostic or therapeutic procedure balloon-assisted colonoscopy can provide an option after failure of conventional colonoscopy. Based on our results and review of the literature, it is successful in >90% of even very difficult cases, and it is safe and well tolerated in a predominantly elderly and comorbid population. When a complete procedure is required in difficult colons for therapy, BAC is an option to consider.

## Figures and Tables

**Table 1:** Indications for balloon-assisted colonoscopy.

Indication	Cases (% of total)
Polyp(s)	53 (43.4)
IBD Surveillance	18 (14.8)
Iron Deficiency Anaemia	16 (13.1)
OGIB/PR Bleeding	14 (11.5)
Suspected Cancer	7 (5.7)
Abnormal Imaging	6 (4.9)
Abdominal Pain	3 (2.5)
Control of Post-Operative Bleeding	2 (1.6)
Diarrhoea	2 (1.6)
Treatment of Angioectasia(s)	1 (0.8)

IBD, Inflammatory bowel disease; OGIB, obscure/occult GI bleeding; PR, per rectum.

**Table 2:** Previous abdominal surgery and comorbidities in patients undergoing balloon-assisted colonoscopy.

Previous Abdominal Surgery	Number (%)	Comorbidities	Number (%)
Hysterectomy (±BSO)	13 (10.7)	Hypertension	32 (26.2)
Gastric Surgery	5 (4.1)	Respiratory	28 (23.0)
Pelvic Floor Repair	4 (3.3)	Cardiac	27 (22.1)
Right Hemicolectomy	4 (3.3)	Diabetes	25 (20.5)
Renal/Adrenal	3 (2.5)	Rheumatology	21 (17.2)
Perforation	3 (2.5)	Functional	13 (10.7)
Appendicectomy	2 (1.6)	Endocrine	12 (9.8)
Meckel’s Diverticulectomy	2 (1.6)	IBD	12 (9.8)
Cystectomy	2 (1.6)	Psychiatric	11 (9.0)
Sigmoid Resection	2 (1.6)	Neurological	11 (9.0)
Splenectomy	1 (0.8)	Other Malignancy	10 (8.2)
Caesarean Section	1 (0.8)	Obesity	10 (8.2)
Prostatectomy	1 (0.8)	Hepato-Pancreatic	9 (7.4)
No Documented Surgery	83 (68.0)	Renal	8 (6.6)
		Vascular	6 (4.9)
		No Major Comorbidity Listed	22 (18.0)

BSO, Bilateral salpingo-oophorectomy; IBD, inflammatory bowel disease.

**Table 3:** Characteristics of those failing BAC.

Characteristic	Number (%)
Age	Median 66.0 (44–86) years
Female	4 (44.4)
Midazolam	Mean 3.4 (±1.5) mg Excluding One Having GA
Fentanyl	Mean 39.3 (±37.8) ug Excluding One Having GA
Unable to Proceed Due to Preparation	2 (22.2)
Obstructing Tumour	1 (11.1)
Previous Surgery	6 (66.7)
Reason for Failed Colonoscopy	Long/Looping/Redundant Colon (7); Discomfort (1- Note Repeated With GA); Fixed Sigmoid (1)
Indication	PR Bleeding (2), Polyps (2), Abdominal Pain (2), IBD (1), Post Op Bleeding (1), Diarrhoea (1)

BAC, balloon-assisted colonoscopy; GA, general anaesthesia; PR, per rectum; IBD, inflammatory bowel disease.

**Table 4:** Common, non-polyp findings. APC, argon plasma coagulation.

Primary Finding of BAC	Procedures (*n* =)	Therapy Required
Colitis/Ileitis	13	Biopsies Only
Diverticulosis	11	Polypectomy for Incidental Polyps (*n* = 5)
Angioectasia	5	APC (*n* = 3), Clip and APC (*n* = 1) and Polypectomy (*n* = 1)
Tumour	4	Biopsies Only
Normal	29	*n*/a

**Table 5:** Summary of available literature. DY, diagnostic yield; *n*/s, not specified.

Authors, Year	Country	Type of Study	No. Of Patients (M/F)	Age: Mean ±SD Or Median (Range in Years)	Type of Endoscope	Caecal Intubation Rate, *n* (%)	Time to Caecum: Mean ±SD Or Median (Range in Min)	DY Of BAC (*n)*	Interventions Performed (*n*)	Adverse Events, (*n*)
May et al., 2006 [13]	Germany	Prospective, Single Centre	14 (6/8)	62 ± 15	Single Balloon	14/14 (100)	*n*/s	Polyps (6) IBD (3) Cancer (2)	Polypectomy (6) Biopsy (5)	Polypectomy Bleeding (1)
Kaltenbach et al., 2006 [14]	USA	Prospective, Single Centre	20 (16/4)	66 ± 12	Single Balloon	19/20 (95)	28 ± 20	Significant (7) (Polyps (5) Ibd (2))	Polypectomy (5) Biopsy (1)	None
Das 2007 [15]	USA	*n*/s, Single Centre	16 (*n*/s)	*n*/s	Double Balloon	14/16 (87.5)	27 ± 9.5	*n*/s	Polypectomy (6) Haemostasis (1)	None
Gay & Delvaux, 2007 [16]	France	Retrospective, Single Centre	29 (5/24)	54±17	Double Balloon	28/29 (96.6)	18 ± 14	Diverticulosis (7) Polyps (4) IBD (2)	Biopsy (8) APC (2) Polypectomy (2)	None
Moönkemuüller et al., 2007 [17]	Germany	*n*/s, Single Centre	7 (3/4)	64(50–75)	Double Balloon	7/7 (100)	15 (9–25)	Diverticulosis (2) Polyps (2) Stenosis (2)	*n*/s	*n*/s
Pasha et al., 2007 [18]	USA	Retrospective, Single Centre	16 (5/11)	69 ± 12	Double Balloon	14/16 (87.5)	27 ± 9.5	*n*/S	Polypectomy (6) APC (1)	*n*/s
Moreels & Pelckmans, 2008 [19]	Belgium	Retrospective, Single Centre	26 (*n*/s)	*n*/s	Double Balloon	23/26 (89)	*n*/s	*n*/S	*n*/s	*n*/s
Moreels et al. 2010 [20]	Belgium	Prospective, Single Centre	45 (28/17)	63 ± 2	Double Balloon	42/45 (93.3)	*n*/s	Polyps (18)	Polypectomy (18) APC (3), Biopsy (2)	None
Teshima et al., 2010 [10]	Netherlands	Prospective, Single Centre	23 (14/8)	53(19–75)	Single Balloon	22/23 (96)	30 (20–60)	Polyps (6) IBD (5) Diverticulosis (2)	Polypectomy (6) Dilation (1)	None
Matsushita et al., 2011 [21]	Japan	Retrospective, Single Centre	24 (*n*/s)	*n*/s	Double Balloon	24/24 (100)	17	*n*/S	17 Interventions (*n*/s)	*n*/s
Keswani et al., 2011 [22]	USA	Prospective, Single Centre	14 (4/10)	58.5 ± 12.5 (35–74)	Single Balloon	13/14 (92.9)	22 ± 18 (10–81)	Polyps (7)	*n*/s	None
Dzeletovic et al., 2012 [23]	USA	Retrospective, Single Centre	53 (12/41)	71(43–83)	Single Balloon& Double Balloon	51/53 (96) (26/26 SBC, 25/27 DBC)	19 (7–58)	Polyps (32) Diverticulosis (21)	Polypectomy (37)	None
Goómez et al., 2012 [24]	USA	Retrospective, Single Centre	45 (21/24)	67(21–84)	Double Balloon	46/51 (90)	*n*/s	Polyps (28) Angiectasia (5)	Polypectomy (28)	None
Hotta et al., 2012 [25]	Japan	Prospective, Multicentre	110 (62/48)	66.5(27–79)	Double Balloon	110/110 (100)	12 (4–47)	Polyps (55) Cancer (5)	Polypectomy (45) Biopsy (8)	Mild Mucosal Injury (1)
Suzuki et al., 2012 [26]	Japan	Prospective, Single Centre	47 (22/25)	63.4 ± 10.9	Double Balloon	47/47 (100)	13.0 ± 5.3	Polyps (21) C ancer (1)	Polypectomy/Resection (6) Further Surgery (1)	None
Kobayashi et al., 2013 [27]	Japan	*n*/sSingle Centre	15 (8/7)	65.7 ± 8.7 (38–81)	Single Balloon	15/15 (100)	22.9 ± 8.9 (9–40)	Polyps Radiation Colitis (3) Diverticulosis (3)	Polypectomy (*N*/S) Biopsy (*N*/S)	None
Yamada et al., 2013 [28]	Japan	Prospective, Single Centre	21 (*n*/s)	71.5 ± 7.8	Single Balloon& Double Balloon	20/21 (95)	12.8 (9.5–42)	Polyps (8) Diverticulosis (7) Cancer (1)	Polypectomy (8) Biopsy (1)	None
Becx & Al-Toma, 2014 [29]	Netherlands	Retrospective, Single Centre	114 (45/69)	64.8(31–91)	Double Balloon	101/114 (88.6)	*n*/s	New Diagnoses (55)	Polypectomy (51) Further Surgery (7)	Bleeding with Spontaneous Resolution (2)
Nemoto et al., 2014 [30]	Japan	Prospective, Single Centre	28 (14/14)	74(35–88)	Double Balloon	28/28 (100)	16 (6–66)	*n*/S	*n*/s	None
Yung et al., 2016 [11]	UK	Retrospective, Single Centre	57 (26/31)	62.9(20–89)	Double Balloon	55/57 (96.5)	*n*/s	29	Polypectomy/APC (22)	None
Sulz et al., 2016 [31]	Switzerland	Retrospective, Single Centre	100 (46/54)	70(38–87)	Single Balloon	98/100 (98)	27.5 (4–92)	Diverticulosis (54) Polyps (47), Colitis (4) Cancer (1)	Polypectomy (45) APC (1)	Mucosal Defect After Polypectomy (1) Haematochezia (1)
Despott et al., 2017 [32]	UK	Prospective, Single Centre	22 (7/15)	68 ± 10	Double Balloon	22/22 (100)	17.5 (16.0–23.7)	*n*/S	*n*/s	*n*/s
Hermans et al., 2018 [33]	Netherlands	Retrospective, Single Centre	61 (34/27)	65(29–82)	Double Balloon	60/63 (95)	*n*/s	Polyps (34) Cancer (3)	Polypectomy (34) Biopsy (3)	Bleeding at Polypectomy (1)
Robertson et al., 2020	UK/Sweden	Retrospective, Multicentre	122 (56/66)	64.4 ± 12.3	Single Balloon& Double Balloon	113/122 (92.6)	20.9 ± 13.5	Polyps (67) , Cancer (4) Colitis (13), Diverticulosis (11) Angiectasia (5)	Polypectomy (60) APC (4) Biopsy	Vasovagal Reaction During Polypectomy (1)
